# Angiotensin-converting enzyme 2 regulates endoplasmic reticulum stress and mitochondrial function to preserve skeletal muscle lipid metabolism

**DOI:** 10.1186/s12944-019-1145-x

**Published:** 2019-11-27

**Authors:** Xi Cao, Xin-Meng Lu, Xiu Tuo, Jing-Yi Liu, Yi-Chen Zhang, Li-Ni Song, Zhi-Qiang Cheng, Jin-Kui Yang, Zhong Xin

**Affiliations:** 1 Beijing Key Laboratory of Diabetes Research and Care, Beijing Diabetes institute, Department of Endocrinology, Beijing Tongren Hospital, Capital Medical University, Beijing, 100730 China; 2 Department of Endocrinology, Beijing Ditan Hospital, Capital Medical University, Beijing, 100015 China; 3 Department of Pharmacology and Molecular Sciences, Johns Hopkins University School of Medicine, Baltimore, MD 21205 USA

**Keywords:** ACE2, Intramuscular fat, Endoplasmic reticulum, Mitochondrial function

## Abstract

**Objective:**

Endoplasmic reticulum (ER) stress and mitochondrial function affected intramuscular fat accumulation. However, there is no clear evident on the effect of the regulation of ER stress and mitochondrial function by Angiotensin-converting enzyme 2 (ACE2) on the prevention of intramuscular fat metabolism. We investigated the effects of ACE2 on ER stress and mitochondrial function in skeletal muscle lipid metabolism.

**Methods:**

The triglyceride (TG) content in skeletal muscle of ACE2 knockout mice and Ad-ACE2-treated db/db mice were detected by assay kits. Meanwhile, the expression of lipogenic genes (*ACCα*, *SREBP-1c*, *LXRα*, *CPT-1α, PGC-1α* and *PPARα*), ER stress and mitochondrial function related genes (*GRP78*, *eIF2α*, *ATF4*, *BCL-2*, and *SDH6*) were analyzed by RT-PCR. Lipid metabolism, ER stress and mitochondrial function related genes were analyzed by RT-PCR in ACE2-overexpression C2C12 cell. Moreover, the IKKβ/NFκB/IRS-1 pathway was determined using lysate sample from skeletal muscle of ACE2 knockout mice.

**Results:**

ACE2 deficiency in vivo is associated with increased lipid accumulation in skeletal muscle. The ACE2 knockout mice displayed an elevated level of ER stress and mitochondrial dysfunctions in skeletal muscle. In contrast, activation of ACE2 can ameliorate ER stress and mitochondrial function, which slightly accompanied by reduced TG content and down-regulated the expression of skeletal muscle lipogenic proteins in the db/db mice. Additionally, ACE2 improved skeletal muscle lipid metabolism and ER stress genes in the C2C12 cells. Mechanistically, endogenous *ACE2* improved lipid metabolism through the IKKβ/NFκB/IRS-1 pathway in skeletal muscle.

**Conclusions:**

ACE2 was first reported to play a notable role on intramuscular fat regulation by improving endoplasmic reticulum and mitochondrial function. This study may provide a strategy for treating insulin resistance in skeletal muscle.

## Introduction

Intramuscular fat is an indispensable energy source for skeletal muscle. These lipids play pivotal roles in metabolism not only for skeletal muscle but also for the entire body. The status of the endoplasmic reticulum (ER) is a significant determinant of protein homeostasis in muscle cells. Accumulation of unfolded proteins and other physiological stresses produces ER stress, which initiates the unfolded protein response (UPR). More importantly, the signaling pathway activated by the ER stress has emerged as a critical regulator of lipid biosynthesis, insulin resistance, inflammation, and apoptosis [1, 2].

Excess lipid accumulation and impairment in mitochondrial function have been considered as putative mechanisms for the pathogenesis of skeletal muscle insulin resistance. Mitochondria modulate the balance between lipid metabolism and storage in the skeletal muscle. β-oxidation of fatty acids (FAs) is linked to ATP production, mitochondrial respiration [3], and redox balance [4]. Muscle mitochondria regulate the transport and β-oxidation flux of long-chain fatty acids [5]. Mitochondrial deficiency as the cause of impaired fatty acid oxidation capacity and skeletal muscle fat accumulation, ultimately leading to the mitochondrial-driven ‘lipotoxicity’ hypothesis of insulin resistance [6].

Renin-angiotensin system (RAS) plays an important role in the pathogenesis of IR [7, 8]. Angiotensin-converting enzyme 2 (ACE2) decreases the generation of Ang II by catalyzing the conversion of Ang II to angiotensin-(1–7) (Ang-(1–7)). Ang-(1–7) elicits the opposite effect of Ang II [9]. Recently, we reported that ACE2 regulates mitochondrial function in pancreatic β-cells, and ACE2 inhibits endoplasmic reticulum stress-associated pathway to preserve hepatic insulin resistance and hepatic steatosis [10, 11]. Moreover, the beneficial protection against ER stress in heart and lung by ACE2 is reported [12, 13]. However, there is no direct evidence that ACE2 regulates ERS and mitochondrial function in the skeletal muscle.

Though ACE2 influences the lipid metabolism of adipose tissue and liver, its effect on intramuscular fat has never been reported. A previous study reported that Ang-(1–7) improved the sensitivity of insulin by skeletal muscle glucose uptake increasement in vivo [14]. However, the underlying mechanism is still unclear. In the present study, we investigated the role of ACE2 in regulating intramuscular fat and its possible behavior on endoplasmic reticulum and mitochondrial function to clarify the function of ACE2 in metabolism and provided a potential target for insulin resistance prevention in skeletal muscle.

## Materials and methods

### Animal

*ACE2* KO mice were a gift from Prof. Josef Penninger from the Institute of Molecular Biotechnology, Austria. In all experiments, the *ACE2* KO mice were identified by PCR genotyping assays from tail biopsies. Only male *ACE2* KO mice *(ACE2*^−/y^ mice) and their age- and sex-matched wild-type (WT) littermates were performed. The male db/db mice at the age of 6 weeks were purchased from Nanjing Biological Medicine Research Institute, Nanjing University, China. The db/db mice were maintained on the BKS background. All mice were maintained on a 12 h light/dark cycle. All animals were handled in accordance with the protocol approved by the Ethics Committee of Animal Research at Beijing Tongren Hospital, Capital Medical University, Beijing, China.

### Biochemical assays

Intramyocellular triglycerides were extracted and determined using a triglyceride assay kit (ThermoFisher Scientific) and normalized to tissue weights.

### Histochemistry

Basic muscle morphology was assessed with haematoxylin and eosin staining, according to a standard protocol. All stained, sectioned images were captured under a microscope with a minimum of four fields of view per muscle section at × 100 magnification.

### Cell culture

Undifferentiated mouse C2C12 myoblasts were maintained in growth medium (GM) (DMEM, Gibco, 11,965–092, supplemented with 10% fetal bovine serum, heat inactivated, 1% penicillin/streptomycin, 1% L-glutamine and 1-mM sodium pyruvate). To prepare for differentiation, the cells were seeded at a density of 1.6 × 10^5^ cells/well in GM in 6-well plates. Differentiation was initiated 2 days later when the cells became confluent by replacing GM with differentiation medium (DM) containing 2% horse serum in place of 10% FBS. The medium was changed every other day until transfection, which was performed on day 4–5 after initiation of differentiation. The cells were maintained at 37 °C with humidified air at 5% CO_2_ and passaged by trypsinization.

### Overexpression of ACE2 in C2C12 cells and mouse liver

To overexpress *ACE2* in the db/db mice, an adenovirus coding for rat *ACE2* (rACE2) upstream of an enhanced green fluorescent protein (eGFP) reporter gene (Ad-rACE2-eGFP) and the control eGFP virus (Ad-eGFP) was respectively injected into the db/db mice (male, 5 to 7-week-old) by tail vein (5 × 10^8^ particle forming units (pfu) in 100 μL saline). On the 6th day post-virus injection, glucose tolerance tests (GTT) were performed. On the 7th day, the animals were sacrificed for experimental analysis. The C2C12 cells were infected with 1.0 × 10^7^ pfu Ad-ACE2 or Ad-GFP for 24 h to overexpress the ACE2 protein.

### Western blot

Total protein was extracted from muscle tissue and C2C12 cells with RadioImmunoprecipitation Assay (RIPA) lysis buffer, and assessed by the BCA protein assay kit for the amount (Beyotime, China). 30–60 μg protein samples were separated by 10% SDS-PAGE and transferred to PVDF membranes (Millipore, Billerica, MA, USA). The membranes were blocked with 5% non-fat dry milk and incubated with antibodies (Abs) at 4 °C overnight. The blots were probed with HRP-conjugated anti-IgG followed by detection with enhanced chemiluminescence (ECL, Millipore). C/EBP homologous protein (CHOP) Ab, activating transcription factor-4 (ATF4) Ab, Bcl-2 Ab, Bax Ab, NDUFB8 Ab, NFκB Ab, phospho-NFκB Ab, IKKβ Ab, phospho-IKKβ Ab, IRS-1 Ab, phospho-IRS-1 (Ser^307^) Ab, and tubulin Ab were purchased from Cell Signaling technology. Glucose regulated protein 78 (GRP78) Ab and eIF2ɑ Ab were obtained from Abcam. acetyl-CoA carboxylase α (ACCα) Ab, liver X receptor-α (LXRα) Ab, sterol regulatory element-binding protein-1c (SREBP-1c) Ab, and (carnitine palmitoyltransferase 1 α) CPT-1α Ab were purchased from Santa Cruz. The 68 kDa band of SREBP-1c was used to calculate the gray value.

### Total RNA extraction and real-time PCR

Total RNA was extracted by Trizol Reagent (Invitrogen). A total of 500 ng of RNA was applied as the template for the first-strand cDNA synthesis by ReverTraAceqPCR RT Kit (TOYOBO, Osaka, Japan). The transcripts were quantified using Light Cycler 480 Real-Time PCR system (Roche, Basel, Switzerland). Primers were designed by Primer Quest (Integrated DNA Technologies, Inc).

### Statistical analysis

All data were presented as the mean ± SD and analyzed by Student’s t-test or one-way ANOVA (with Bonferroni post-hoc tests to compare replicate means) when appropriate. Statistical comparisons were performed by Prism5 (GraphPad Software, San Diego, CA). *P* value less than 0.05 were identified to be statistically significant. Representative results from at least three independent experiments were shown unless otherwise stated.

## Results

### ACE2 deficiency in vivo displayed lipid accumulation, ER stress and mitochondrial dysfunction in skeletal muscle

**Fig. 1:**
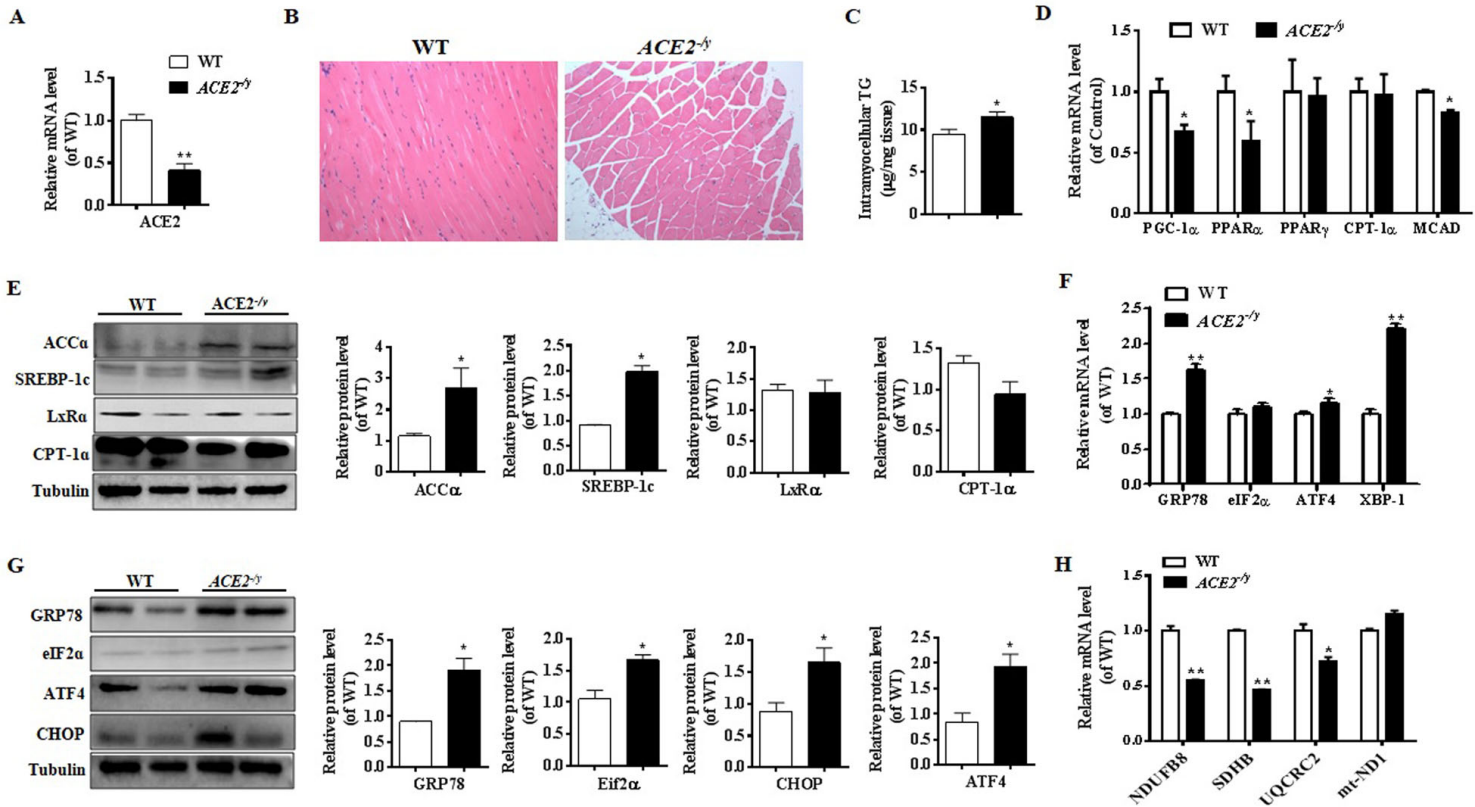
Analysis of lipid metabolism, ER stress and mitochondrial function in skeletal muscle of *ACE2*^*−/y*^ mice. **a**: Relative *ACE2* expression level in skeletal muscle of *ACE2*^*−/y*^ mice. **b**: H&E-stained histology of gastrocnemius muscle in the *ACE2*^*−/y*^ and WT mice (100× H&E). **c**: Analysis of intramuscular triglyceride concentration in skeletal muscle of *ACE2*^*−/y*^ mice. **d**: Relative gene expression levels of fatty acid oxidation-related genes (*PGC-1α*, *PPARα*, *PPARγ*, *CPT-1α*, and *MCAD*). **e**: Relative protein levels of lipid-metabolizing (ACCα, SREBP-1c, LXRα and CPT-1α). **f**: Relative gene expression levels of ER stress-related genes (*GRP78*, *Eif2α*, *ATF4* and *XBP-1*). **g**: Relative protein levels of GRP78, Eif2α, ATF4 and CHOP in the skeletal muscle of *ACE2*^*−/y*^ mice. **h**: Relative gene expression levels of NDUFB8, SDHB, UQCRC2 and mt-ND1 in the skeletal muscle of *ACE2*^*−/y*^ mice. The data are presented as the mean ± SD of *n* = 4 independent experiments in *ACE2*^−/y^ mice. *P < 0.05 and **P < 0.01 versus WT mice by Student’s t test

ACE2 knockout mice (*ACE2*^−/y^) were used to evaluate its role in skeletal muscle in vivo. The *ACE2* mRNA levels were indeed reduced in the skeletal muscle of the *ACE2*^−/y^ mice (Fig. 1a). Pathological changes were observed in H&E stained *ACE2*^−/y^ mice skeletal muscle sections. The *ACE2*^*−/y*^ mice exhibited breakage of fibers and disorder of morphology (Fig. 1b).

The skeletal muscle TG content was significantly higher in *ACE2*^−/y^ mice than the WT mice (Fig. 1c). To further explore these findings, we investigated the expression levels of proteins involved in lipid metabolism using real-time PCR and Western blot. Consistently, the mRNA levels of fatty acid oxidation-related genes, including *PPARγ coactivator 1α* (*PGC-1α*), *peroxisome proliferator-activated receptor alpha* (*PPARα*), and *medium chain acy -CoA dehydrogenase* (*MCAD*) were down-regulated, and little change was observed in *PPAR gamma* (*PPARγ*) and *CPT-1α* in the skeletal muscle of the *ACE2*^−/y^ mice (Fig. 1d). Coinstantaneous, the protein levels of lipid-metabolizing genes, including ACCα and SREBP-1c were up-regulated, whereas the expression of LXRα and CPT-1α did not exhibit an obvious difference between the *ACE2*^−/y^ and the WT groups (Fig. 1e). These results suggested that deletion of ACE2 may aggravate intramuscular fat accumulate in the *ACE2*^−/y^ mice.

Next, we examined the ER stress in the skeletal muscle. During the ER stress, several specific proteins were highly expressed, including GRP78, pancreatic endoplasmic reticulum kinase-eukaryotic translation initiation factor 2α (eIF2α), binding immunoglobulin protein (BiP), also known as inositolrequiring enzyme1α (IRE 1α)-X-box-binding protein-1 (XBP-1), activating transcription factor 4 (ATF4), and CHOP. In this study, the mRNA levels of *GRP78*, *ATF4* and *XBP-1* were increased in the *ACE2* knockout mice (Fig. 1f). Consistently, the protein levels of GRP78, eIF2α, ATF4, and CHOP were all significantly up-regulated in the skeletal muscle of the *ACE2*^−/y^ mice (Fig. 1g). These results suggested a progressive accumulation of unresolved ER stress in the skeletal muscle of the *ACE2*^*−/y*^ mice.

To study whether the mitochondrial function affect the skeletal muscle lipid metabolism, the gene levels of mitochondrial complexes I- III were measured. As expected, the mRNA levels of *NDUFB8* (Complex I), succinate dehydrogenase subunit B (*SDHB*) (Complex II), and ubiquinol-cytochrome c reductase complex core protein 2 (*UQCRC2*) (Complex III) were down-regulated significantly (Fig. 1h), whereas no observable difference was detected in mitochondrial encoded NADH dehydrogenase 1 (mt-*ND1*) group. These results indicated that the endogenous *ACE2* may regulate the expressions of proteins related to ER stress, mitochondrial function and cell apoptotic in mice skeletal muscle.

### ACE2 improves skeletal muscle lipid metabolism in vitro and in vivo

**Fig. 2:**
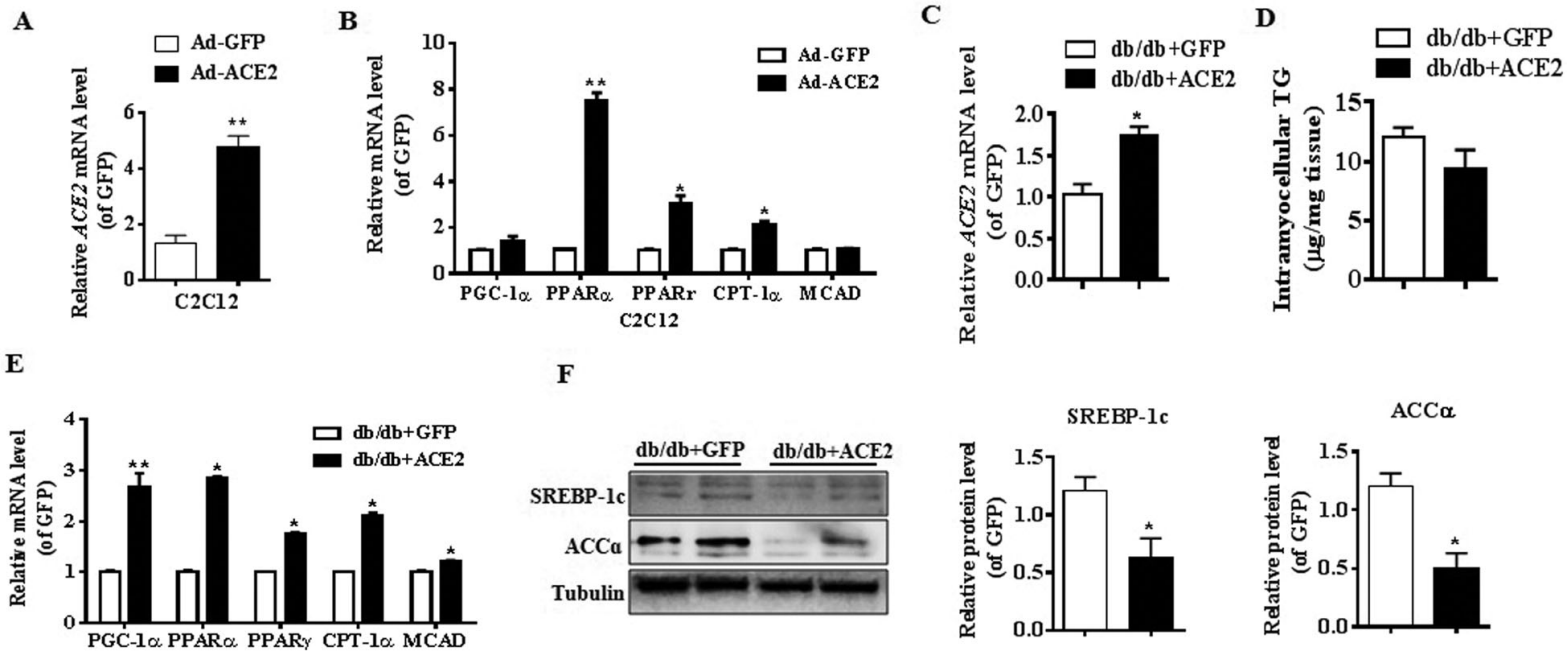
Analysis of lipid metabolism in ACE2-overexpressing C2C12 cells and Ad-ACE2-treated db/db mice. **a**: Relative *ACE2* expression level in ACE2-overexpressing C2C12 cells. **b**: Relative gene expression levels of fatty acid oxidation-related genes (*PGC-1α*, *PPARα*, *PPARγ*, *CPT-1α*, and *MCAD*). **c**: Relative *ACE2* expression level in skeletal muscle of Ad-ACE2-treated db/db mice. **d**: Analysis of intramuscular triglyceride concentration in skeletal muscle of Ad-ACE2-treated db/db mice. **e**: Relative gene expression levels of fatty acid oxidation-related genes (*PGC-1α*, *PPARα*, *PPARγ*, *CPT-1α*, and *MCAD*). **f**: Relative protein levels of lipid-metabolizing (SREBP-1c and ACCα). The data are presented as the mean ± SD of *n* = 3 independent experiments in C2C12 cells, n = 3 in Ad-ACE2-treated db/db mice. *P < 0.05 and **P < 0.01 versus Ad-GFP cell or Ad-GFP-treated mice by Student’s t test

ACE2 was overexpressed in the C2C12 cells to evaluate its role in lipid metabolism and ER stress by RT-PCR analysis in vitro. Firstly, ACE2 has been proved to be indeed overexpressed after the adenoviruses infect (Fig. 2a). Secondly, the mRNA levels of fatty acid oxidation-related genes, *PPARα, PPARγ,* and *CPT-1α* were increased, and little change was observed in *PGC-1α* and *MCAD* in ACE2-overexpressing C2C12 cells (Fig. 2b). The results showed that the overexpression of ACE2 significantly improved fatty acid oxidation and ER stress.

ACE2 was overexpressed by adenovirus in the db/db mice to evaluate its role in skeletal muscle lipid metabolism in vivo. After injection of adenoviruses, RT-PCR result indicated the protein level of ACE2 was overexpressed (Fig. 2c). The content of TG in skeletal muscle of the Ad-ACE2-treated mice was slightly lower than that of Ad-GFP-treated mice (Fig. 2d). The mRNA levels of fatty acid oxidation-related genes, *PGC-1α*, *PPARα*, *PPARγ, CPT-1α,* and *MCAD* were up-regulated in the skeletal muscle of the Ad-ACE2-treated mice compared to the Ad-GFP-treated mice (Fig. 2e). Moreover, the Ad-ACE2-treated mice exhibited a significant reduction of protein level of SREBP-1c and ACC*α* in the skeletal muscle (Fig. 2f).

### ACE2 ameliorates ER stress and mitochondrial function in vitro and in vivo

**Fig. 3:**
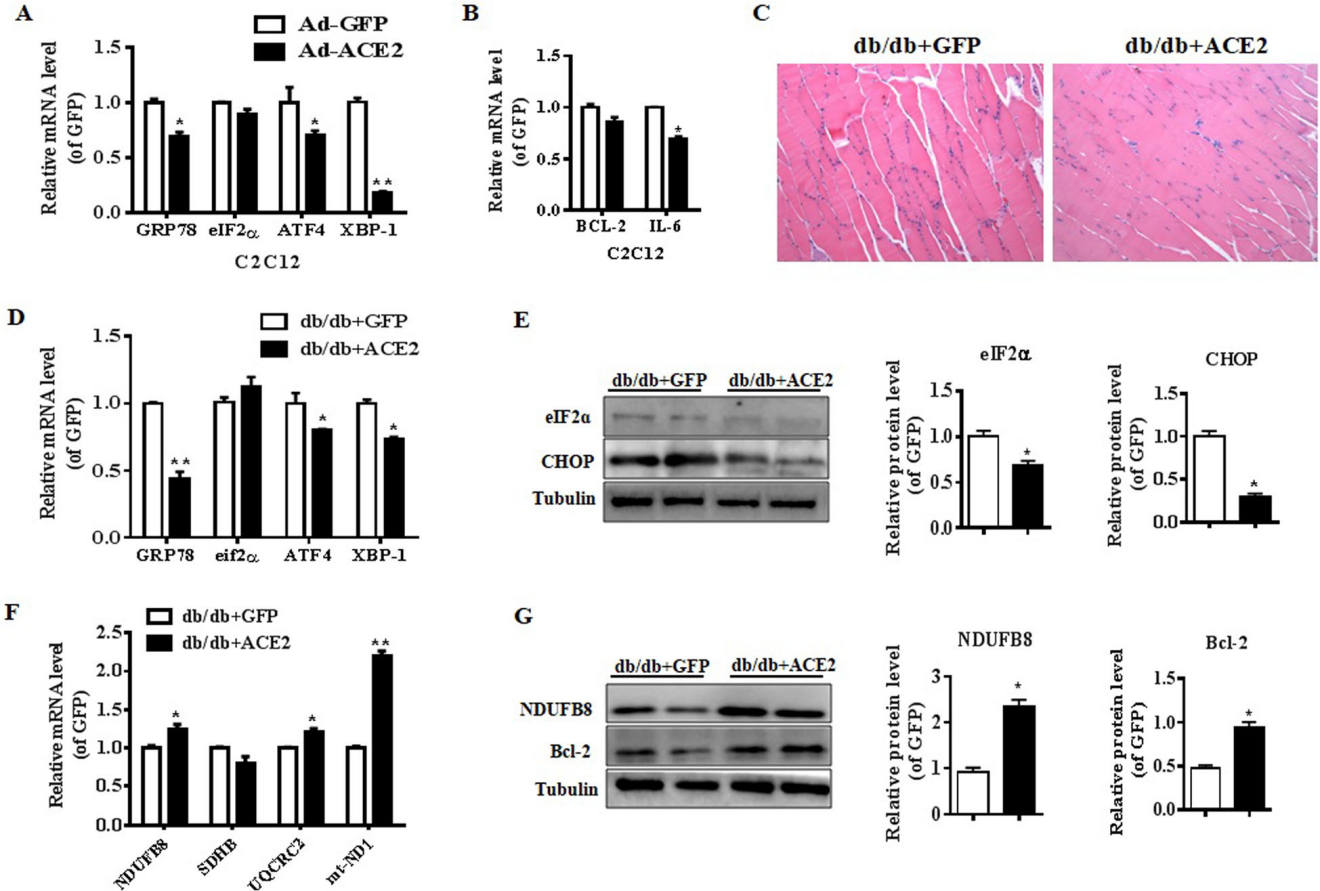
ACE2 regulated ER stress and mitochondrial function in ACE2-overexpressing C2C12 cells and Ad-ACE2-treated db/db mice. **a**: Relative gene expression levels of ER stress-related genes (*GRP78*, *Eif2α*, *ATF4* and *XBP-1*) in ACE2-overexpressing C2C12 cells. **b**: Relative gene expression levels of *BCL-2* and *IL-6* in ACE2-overexpressing C2C12 cells. **c**: H&E-stained histology of gastrocnemius muscle in the Ad-ACE2-treated and Ad-GFP-treated db/db mice (100× H&E). **d**: Relative gene expression levels of ER stress-related genes (*GRP78*, *Eif2α*, *ATF4* and *XBP-1*). **e**: Relative protein levels of eIF2α and CHOP in the skeletal muscle of Ad-ACE2-treated db/db mice. **f**: Relative gene expression levels of NDUFB8, SDHB, UQCRC2 and mt-ND1 in the skeletal muscle of Ad-ACE2-treated db/db mice. **g**: Relative protein levels of NDUFB8 and Bcl-2 in the skeletal muscle of Ad-ACE2-treated db/db mice. The data are presented as the mean ± SD of n = 3 independent experiments in C2C12 cells, n = 3 in Ad-ACE2-treated db/db mice. *P < 0.05 and **P < 0.01 versus Ad-GFP cell or Ad-GFP-treated mice by Student’s t test

In the C2C12 cell, the mRNA levels of *GRP78*, *ATF4* and *XBP-1* were significantly decreased in the ACE2-overexpressing cells, but not *eIF2α* (Fig. 3a). Inflammation gene *IL-6* was also reduced in the ACE2-overexpressing C2C12 cells (Fig. 3b).

Meanwhile, pathological changes were observed in the H&E stained skeletal muscle sections. The Ad-ACE2-treated mice demonstrated less structural damage and consistent protection morphology compared to the Ad-GFP-treated mice (Fig. 3c). Next, we studied whether ACE2 altered the expression of ER stress and mitochondrial function-related genes. We found the mRNA levels of *GRP78*, *ATF4*, *XBP-1* was depressed (Fig. 3d) and the protein levels of eIF2α and CHOP were inhibited in the Ad-GFP-treated db/db *mice* (Fig. 3e)*.* Moreover, the mRNA levels of *NDUFB8*, *UQCRC2,* and mt-*ND1* were increased in the skeletal muscle of the Ad-ACE2-treated mice (Fig. 3f). Additionally, the protein levels of NDUFB8 and Bcl-2 were reduced obviously in the Ad-GFP-treated mice (Fig. 3g). These data indicated ACE2 may significantly improve the skeletal muscle lipid metabolism in the db/db mice. These studies also suggested that ACE2 may regulate ER stress and mitochondrial function in the skeletal muscle.

### ACE2 regulates the IKKβ/NFκB/IRS-1 pathway in muscle

**Fig. 4:**
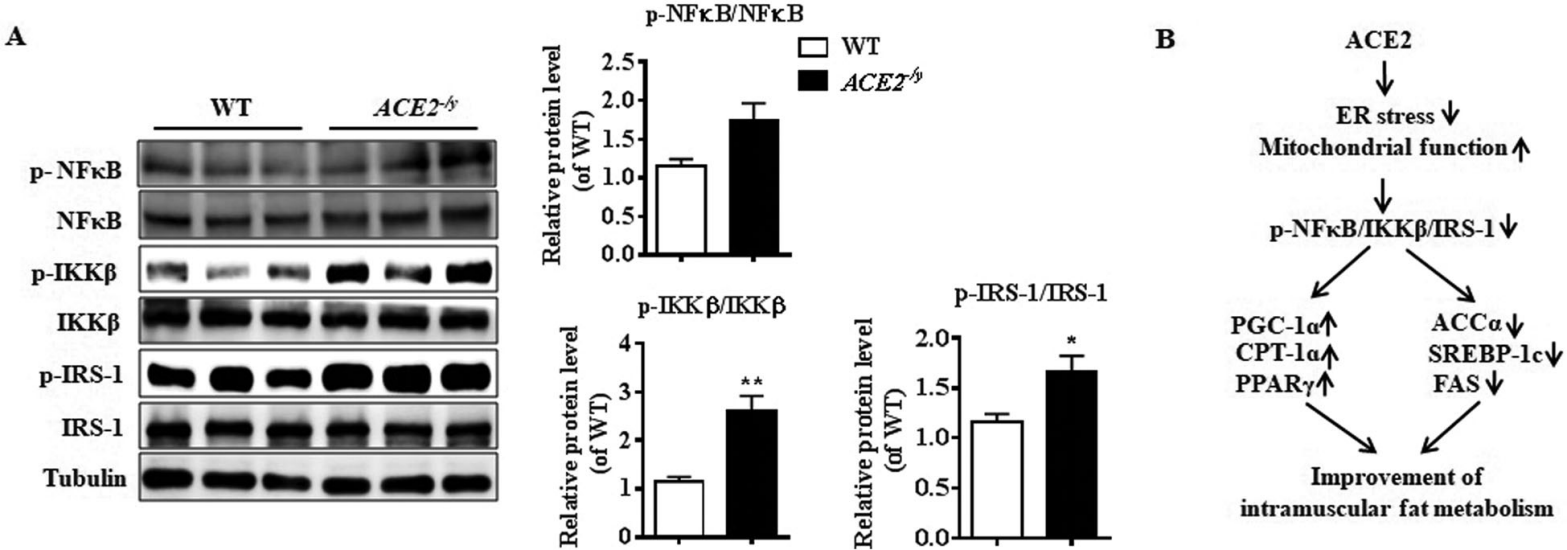
Analysis of IKKβ/NFκB/IRS-1 pathway in skeletal muscle of *ACE2*^−/y^ mice. **a**: Relative protein levels of NFκB (p-NFκB), IKKβ (p-IKKβ), and IRS-1 (Ser307) in skeletal muscle of *ACE2*^*−/y*^ mice. **b**: Proposed model of intramuscular fat metabolism amelioration by the ACE2. The up- or down-regulation of metabolic pathways is indicated by arrows. (↑ for up-regulation and ↓ for down-regulation). The data are presented as the mean ± SD of n = 3 in *ACE2*^*−/y*^ mice. *P < 0.05 versus WT by Student’s t test

To explore a possible mechanism involvement of ACE2 in its beneficial effect against ER stress, we evaluated the expression of the IKKβ/NFκB pathway in the *ACE2*^−/y^ mice and found the levels of phosphorylated NFκB and IKKβ markedly increased. Although the total amount of NFκB and IKKβ protein did not change (Fig. 4a). Consistently, IRS-1, which is another important marker of ER stress downstream of NFκB, was also significantly up-regulated in the *ACE2*^−/y^ mice (Fig. 4a). These results suggested that ACE2-regulated ER stress in the skeletal muscle was also dependent on the IKKβ/NFκB/IRS-1 signaling pathway as in the liver.

## Discussion

This study provided a novel avenue to treat skeletal muscle disease and even other ER stress or mitochondrial-associated pathologies. We demonstrated the amount of lipid accumulation was increased in the skeletal muscle of the ACE2 knockout mice, and the expression of ER stress and mitochondrial function related proteins in skeletal muscle were affected by the lack of ACE2. Moreover, ACE2 up-regulation improved skeletal muscle lipid metabolism and ER stress genes in the C2C12 cells. Importantly, ACE2 may significantly ameliorate skeletal muscle lipid metabolism, ER stress and mitochondrial function in the Ad-ACE2-treated db/db mice.

Increasing fatty acid oxidation while reducing intramuscular lipids is considered as an effective mean to improve insulin sensitivity. PGC-1α has been implicated in the regulation of skeletal muscle oxidative metabolism and mitochondrial biogenesis. As a transcriptional co-activator, PGC-1α activates several transcription factors to drive transcription of vast gene networks involved in many aspects of energy homeostasis, including glucose utilization and fatty acid oxidation, as well as mitochondrial biogenesis and function [15]. Some key transcriptional regulators, such as LXRα and SREBP-1c, coordinately control the lipogenesis, which increase the expression of key lipogenic genes, including those for FAS, SCD1 and ACC [16]. ACC1 converts acetyl-CoA to malonyl-CoA and inhibits fatty acid entry into the mitochondria reducing β -oxidation. In this study, we illustrated that skeletal muscle TG contents were significantly higher in the *ACE2*^−/y^ mice than that in the WT mice. Subsequently, we also found the mRNA levels of fatty acid oxidation genes *PGC-1α*, *PPARα, PPARγ, CPT-1α* and *MCAD* decreased in the ACE2 KO mice, and increased in the ACE2-overexpressing C2C12 cells and the db/db mice. Meanwhile, the protein levels of lipogenesis proteins ACCα and SREBP-1C increased in the ACE2 KO mice, and decreased in the ACE2-overexpressing db/db mice. Accordingly, these results suggested that ACE2 may regulate intramuscular fat accumulate in mice.

It demonstrated the alterations in mitochondria and ER physical interactions contribute to insulin resistance [17]. Indeed, mitochondria and ER interact at contact points, called mitochondria-associated endoplasmic reticulum membranes, in order to exchange calcium (Ca^2+^) and lipids, thus regulating cell metabolism and fate [15]. In consequence of ER stress, an adaptive process named UPR is triggered. Three distinct branches of the UPR system can be initiated by transmembrane effector signal transduction proteins-protein kinase R (PKR)-like ER kinase (PERK), inositol requiring enzyme 1 alpha (IRE1α), and activating transcription factor 6 (ATF6). The UPR consists of three signaling branches which are initiated by signals such as the dissociation of BiP (GRP78) from the intracellular receptor domains of the ER. These signals activate combinations of the three stress sensors, protein kinase RNA-like PERK, ATF6 and inositol-requiring enzyme 1 α (IRE 1α). CHOP is a downstream effector of all the three branches of the UPR [18]. Enhanced CHOP expression has several effects on cells including alteration of the balance of pro- and anti-apoptotic proteins that act on mitochondria [18]. In RAS, Ang II and ACE2 have been reported to induce ER stress via GRP78/eIF2α/ATF4/CHOP axis in the liver [10, 19]. In the present study, the changes in GRP78/eIF2α/XBP-1/ATF4/CHOP expression suggested that ACE2 ameliorates ER stress in skeletal muscle and liver may through the same pathway. The mechanism underlying this process could involve the ability of ACE2 to regulate GRP78/ eIF2α/ XBP-1/ATF4/CHOP pathway.

Mitochondria regulate energy homeostasis by metabolizing nutrients, generating ATP and heat. In skeletal muscle, substantial pieces of evidence show that mitochondrial dysfunctions, in terms of number and functionality, are jointly liable for IR [20, 21]. The respiratory electron transport chain complexes I-IV which transfer the electron in the inner mitochondria membrane and the ATP-synthase enzyme (Complex V) play a critical role in mitochondrial oxidation [22]. Here, we showed that SDHB, NDUFB8 and UQCRC2 were down-regulated in the ACE2 KO mice. Consistently, NDUFB8 and UQCRC2 were down-regulated in the Ad-ACE2-treated db/db mice. These data suggested that ACE2 can improve skeletal muscle mitochondria function by regulating respiratory electron transport chain complexes.

Activation of the pro-inflammatory transcription NF-κB has been connected to the impairment of insulin signaling induced by the ER stress in skeletal muscle [23]. The UPR is mainly mediated by the activation of IRE-1α, PERK/ eIF2-α, and ATF6 signaling pathways. These three cellular pathways can interact with several inflammatory signals, including NF-κB [24]. It has been reported that UPR reduces the NF-κB inhibitor IκBα through several mechanisms [25, 26], which results in NF-κB activation and the regulation of genes involved in inflammation and insulin resistance [24]. Given that accumulation of intracellular lipid species can activate cellular signaling cascades conducive to NFκB activation [27], we check the IKKβ/NFκB signal pathway. Of interest, ACE2 down-regulation resulted in the increased expression of the phosphorylation of IKKβ and NFκB in the skeletal muscle of the ACE2 KO mice. The insulin-signaling pathway requires insulin receptor substrates (IRS-1 and IRS-2). IRS-1 has a major role in skeletal muscle [28]. Serine hyperphosphorylation of IRS-1 is a marker of IR in peripheral tissues, which leads to subsequent activation of PI3K pathway [29]. Akt is a key protein involved in PI3K pathway, its phosphorylation promotes the metabolic activities in skeletal muscle [30]. It’s remarkable that ACE2 down-regulation increased expression of the phosphorylation of IRS-1 in the skeletal muscle of the ACE2 KO mice. However, one previous study showed that phosphorylated Akt in the soleus muscle equally in the standard diet-fed ACE2KO mice compared with the WT mice [31]. These findings suggest that ACE2 mediate ER stress and mitochondria function in intracellular lipid metabolism, which was involved in the regulation of the IKKβ/NFκB/IRS-1 pathway, but not Akt pathway.

## Conclusions

As shown in Fig. 4b, ACE2 ameliorates intramyocellular lipid metabolism could involve the ability of regulating ER stress and mitochondria function in skeletal muscle. The underlying mechanism is mediated partly through the activation of NFκB/ IKKβ/IRS-1 signaling pathway. This study may further provide a strategy for treating insulin resistance in skeletal muscle.

## Data Availability

All data generated or analyzed during this study are included in this published article.

## Abbreviations

ACCα: Acetyl-CoA carboxylase α
ACE2: Angiotensin-converting enzyme 2
ACE2^−/y^: ACE2 knockout mice
Ang-(1–7): Angiotensin-(1–7)
ATF4: Activating transcription factor-4
ATF6: Activating transcription factor 6
BiP: Binding immunoglobulin protein
CHOP: C/EBP homologous protein
CPT-1α: Carnitine palmitoyltransferase 1 α
DM: Differentiation medium
eIF2α: eukaryotic translation initiation factor 2α
ER: Endoplasmic reticulum
FAs: Fatty acids
GM: Growth medium
GRP78: Glucose regulated protein 78
GTT: Glucose tolerance tests
IRE 1α: Inositolrequiring enzyme1 α
IRE1α: Inositol requiring enzyme 1 alpha
LXRα: Liver X receptor-α
MCAD: Medium chain acy-CoA dehydrogenase
mt-ND1: mitochondrial encoded NADH dehydrogenase 1
PERK: Pancreatic endoplasmic reticulum kinase
PGC-1α: PPARγ coactivator 1α
PPARα: Peroxisome proliferator-activated receptor alpha
PPARγ: Peroxisome proliferator-activated receptor gamma
RAS: Renin-angiotensin system
SDHB: Succinate dehydrogenase subunit B
SREBP-1c: Sterol regulatory element-binding protein-1c
TG: Triglyceride
UPR: Unfolded protein response
UQCRC2: Ubiquinol-cytochrome c reductase complex core protein 2
WT: Wild-type
XBP-1: X-box-binding protein-1
